# Elabela, a Novel Peptide, Exerts Neuroprotective Effects Against Ischemic Stroke Through the APJ/miR-124-3p/CTDSP1/AKT Pathway

**DOI:** 10.1007/s10571-023-01352-6

**Published:** 2023-04-27

**Authors:** Kang-long Zhang, Shuang-mei Li, Jing-yu Hou, Ying-hui Hong, Xu-xiang Chen, Chang-qing Zhou, Hao Wu, Guang-hui Zheng, Chao-tao Zeng, Hai-dong Wu, Jia-ying Fu, Tong Wang

**Affiliations:** 1 Department of Emergency, The Eighth Affiliated Hospital of Sun Yat-Sen University, Shenzhen, 518003 Guangdong People’s Republic of China; 2 Department of Emergency, Sun Yat-Sen Memorial Hospital of Sun Yat-Sen University, Guangzhou, 510120 Guangdong People’s Republic of China

**Keywords:** Elabela, Apoptosis, Axon damage, miR-124-3p, C-terminal domain small phosphatase 1, Phosphorylation of AKT

## Abstract

Elabela (ELA), which is the second endogenous peptide ligand of the apelin receptor (APJ) to be discovered, has been widely studied for potential use as a therapeutic peptide. However, its role in ischemic stroke (IS), which is a leading cause of disability and death worldwide and has limited therapeutic options, is uncertain. The aim of the present study was to investigate the beneficial effects of ELA on neuron survival after ischemia and the underlying molecular mechanisms. Primary cortical neurons were isolated from the cerebral cortex of pregnant C57BL/6J mice. Flow cytometry and immunofluorescence showed that ELA inhibited oxygen–glucose deprivation (OGD) -induced apoptosis and axonal damage in vitro. Additionally, analysis of the Gene Expression Omnibus database revealed that the expression of microRNA-124-3p (miR-124-3p) was decreased in blood samples from patients with IS, while the expression of C-terminal domain small phosphatase 1 (CTDSP1) was increased. These results indicated that miR-124-3p and CTDSP1 were related to ischemic stroke, and there might be a negative regulatory relationship between them. Then, we found that ELA significantly elevated miR-124-3p expression, suppressed CTDSP1 expression, and increased p-AKT expression by binding to the APJ receptor under OGD in vitro. A dual-luciferase reporter assay confirmed that CTDSP1 was a direct target of miR-124-3p. Furthermore, adenovirus-mediated overexpression of CTDSP1 exacerbated neuronal apoptosis and axonal damage and suppressed AKT phosphorylation, while treatment with ELA or miR-124-3p mimics reversed these effects. In conclusion, these results indicated that ELA could alleviate neuronal apoptosis and axonal damage by upregulating miR-124-3p and activating the CTDSP1/AKT signaling pathway. This study, for the first time, verified the protective effect of ELA against neuronal injury after ischemia and revealed the underlying mechanisms. We demonstrated the potential for the use of ELA as a therapeutic agent in the treatment of ischemic stroke.

**Graphical Abstract:**

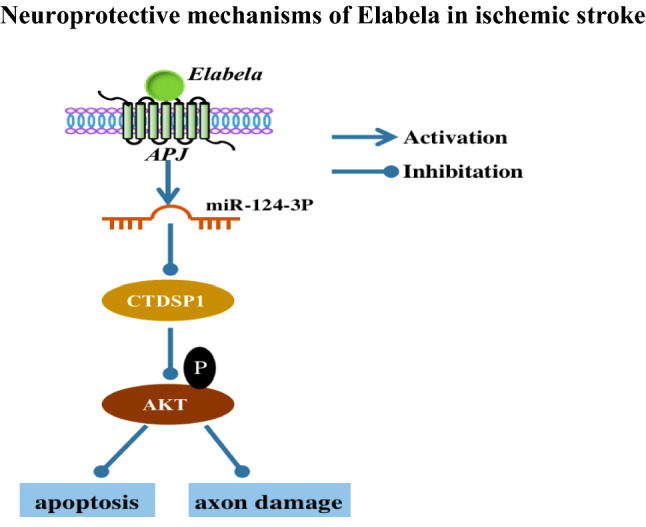

**Supplementary Information:**

The online version contains supplementary material available at 10.1007/s10571-023-01352-6.

## Introduction

Ischemic stroke (IS) is a cerebrovascular disease that is associated with high morbidity, mortality, disability, and recurrence rates; IS poses a serious threat to human health, and there are limited therapeutic options (Saini et al. 2021; Feigin et al. 2017). Neuronal loss caused by ischemia and infarcts is one of the most direct causes of cerebral functional deficits (Mendelson and Prabhakaran 2021). Recent research has revealed that many neurons in the ischemic penumbra or peri-infarct zone may undergo apoptosis after several hours or days, and there is an opportunity to salvage these neurons via poststroke treatment (Uzdensky 2020). On the other hand, axon damage can inhibit signal transmission, thereby contributing to the pathology of IS and resulting in brain dysfunction. Even if neurons survive, axonal damage still prevents the recovery of brain function (Huang et al. 2021). Researchers have discovered that the survival of injured neurons is a necessary prerequisite for axonal regrowth (Guo et al. 2021; Zhou et al. 2016). Hence, the development of neuroprotective drugs that target and prevent apoptosis and axon damage in penumbra neurons seems to be a rational and necessary therapeutic goal for the treatment of ischemia.

Elabela (ELA) is a recently discovered endogenous peptide ligand of the apelin receptor (APJ), and it has been widely studied as a potential therapeutic peptide due to its safety and efficacy (Zhang et al. 2018; Chng et al. 2013). Previous studies have found that the ELA-APJ axis is essential for diverse biological processes, and this axis has been shown to regulate fluid homeostasis, myocardial contractility, vasodilation, angiogenesis, cellular differentiation, apoptosis, oxidative stress, cardiorenal fibrosis, and dysfunction (Ma et al. 2021). Systemic administration of ELA exerts vasodilatory, antihypertensive, cardioprotective, and renoprotective effects and prevents the pathogenesis of preeclampsia (Xu 2021). Moreover, many studies have shown that APJ is widely expressed in the central nervous system, especially in neurons (Luo et al. 2020). Apelin can cross the blood‒brain barrier (BBB), and the apelin/APJ axis exerts neuroprotective effects by inhibiting neuronal apoptosis and improving functional recovery through diverse pathways during IS (Tian et al. 2020). Moreover, mature ELA peptides, including ELA-32, have comparable potency and similar structure to pyroglutamate apelin-13 [(Pyr1)-Apelin-13] (Yang et al. 2017), which means that ELA may also cross the BBB and play a neuroprotective role by activating the APJ receptor.

MicroRNAs (miRNAs) are endogenous small-molecule noncoding RNAs that can exert their effects by binding to the 3′ untranslated regions (3′UTRs) of specific target mRNAs to promote the degradation of these target mRNAs and/or inhibit their translation. MiRNA levels in the blood and brain have been reported to be altered after stroke in both rodents and humans (Li et al. 2018; Saugstad 2015). Furthermore, miRNAs can be modulated by external agents to improve functional outcomes after stroke (Wu et al. 2019). MiR-124 is one of the most abundant miRNAs in brain tissues. An increasing number of studies have demonstrated that miR-124 is aberrantly expressed in peripheral blood and brain cells after cerebral ischemia and that it extensively participates in the pathophysiology following IS, suggesting that miR-124 is a promising candidate biomarker for time-sensitive diagnosis and a therapeutic target of IS (Liu et al. 2019). Furthermore, analysis of data from the GEO database revealed that the expression of miR-124-3p was decreased in blood samples from patients with IS, while the expression of CTDSP1 was increased, indicating that the levels of both miR-124-3p and CTDSP1 were altered under ischemic conditions. However, it is unclear whether miR-124-3p participates in oxygen-glucose deprivation (OGD)-induced neuronal injury by targeting CTDSP1.

CTDSP1, which is an aldiphosphatase enzyme, has been found to participate in various cellular activities, such as neuronal gene silencing, cell cycle regulation, and cell signal transduction (Rallabandi et al. 2020). Gervasi found that CTDSP1 knockdown promoted neurotrophic factor expression in both dorsal root ganglion (DRG) neurons and support cells and promoted DRG neuron axon growth (Gervasi et al. 2021). Furthermore, Liao found that CTDSP1 could localize to the plasma membrane and specifically dephosphorylate AKT at S473, which inactivated AKT and negatively regulated angiogenesis (Liao et al. 2017). Akt is a critical survival factor that has been implicated in brain development, aging, and disease. Decreased expression of Akt is associated with neurodegeneration, and the activation of Akt is neuroprotective (Zhao et al. 2016). Importantly, accumulating data have shown that AKT can be activated by phosphorylation at S473 by a large variety of molecules, and activated AKT can relay signals to regulate downstream neuron apoptosis and axon growth (Liu et al. 2022). Our previous discoveries also showed that ELA could promote anti-apoptosis processes in mesenchymal stem cells (MSCs) under hypoxic/ischemic conditions by activating the PI3K/AKT signaling pathways in an APJ-dependent manner (Fu et al. 2020).

At present, no research has focused on the regulatory effect of ELA on neuronal survival after IS. Here, we explored the role of ELA in ischemia neurons and the related underlying mechanism to facilitate its application in clinical practice.

## Materials and Methods

### Animals

C57BL/6J mice (female, Embryonic 16–18 days, SPF grade) were purchased from the Zhuhai BesTest Bio-Tech Co., Ltd (Zhuhai, China). Before any experiments, all animals were kept in quarantine for 16–18 days in individually ventilated cages with optimum water and food (normal pellet diet). All the animal experiments were approved by the Animal Ethics Committee of the Eighth Affiliated Hospital of Sun Yat-sen University (2023-003-01).

### Chemicals

ELA, which is a polypeptide that is composed of 32 amino acids (sequence: QRPVNLTMRRKLRKHNCLQR RCMPLHSRVPFP), was synthesized by GL Biochem Shanghai Ltd. (China). The ELA powder was 95.31% pure and stored at − 20 °C. Before utilization, ELA was dissolved in PBS and sterilized with a 0.22-µm filter. The concentration of ELA that was used in this study was 1 µM.

### Cell Isolation and Culture

Primary cortical neuron cultures were prepared from embryonic day 16–18 (E16-18) mouse brains. Cortices were digested with 0.25% trypsin (Beyotime, China) for 15 min at 37 °C under sterile conditions and then, the reaction was stopped by incubation with an equal volume of plating medium supplemented with 10% fetal bovine serum (Thermo Fisher, USA) for 5 min at room temperature. The tissues were triturated by gently pipetting in plating medium consisting of MEM without glutamine (GIBCO, USA), 10% FBS (Thermo Fisher, USA), 1 mM l-glutamine (Invitrogen, USA), 10-mM HEPES (GIBCO, USA), and 50-units/mL antibiotics (penicillin and streptomycin) (HyClone, USA) until fully dissociated. The cells were diluted to an appropriate concentration and plated in 6-cm dishes that were precoated with polylysine (Solarbio, China) and laminin (Sigma-Aldrich, USA). Three hours later, the cells were grown in culture medium consisting of neurobasal medium (Thermo Fisher, USA), 2% B27 supplement (GIBCO, USA), 0.5-mM l-glutamine (Invitrogen, USA), and 50-units/mL antibiotics (penicillin and streptomycin) (HyClone, USA) (Kaech and Banker 2006).

### Data Collection

The IS data were obtained from Gene Expression Omnibus (GEO) (https://www.ncbi.nlm.nih.gov/geo/). The data (accession number: GSE95204) from 3 IS samples and 3 normal samples were downloaded and used to analyze the differential expression of miR-124-3p. Moreover, the differences in CTDSP1 expression between the IS and normal samples were also analyzed with data from the GEO database (accession numbers: GSE122709 and GSE16561).

### Flow Cytometry Assay

The Annexin V–FITC/PI apoptosis detection kit (BD, USA) was used to assess the apoptosis of neurons according to the standard protocol. After the designated treatment, cells were harvested with 0.25% trypsin (without EDTA) and centrifuged at 1000 rpm for 5 min. Subsequently, neurons were washed twice with cold PBS and resuspended in 100 μL 1 × binding buffer. Five microliters of Annexin V–FITC and 5 μL of PI were added and gently mixed in each tube. Neurons were then incubated in the dark at room temperature for 15 min before adding 400 μL of 1 × binding buffer. Finally, cell samples were detected using flow cytometry (BD LSRFortessa, USA) within an hour and analyzed using FlowJo software.

### Evaluation of Axon Growth by Immunofluorescence Staining

Neurons were fixed with 4% paraformaldehyde for 30 min, permeabilized with PBS/0.5% Triton for 5 min, blocked with PBS/5% BSA and incubated at 4 °C with a rabbit anti-β III tubulin (1:500, ABCAM, USA) primary antibody overnight. A polyclonal goat anti-rabbit IgG (H + L) secondary antibody [FITC] was diluted in PBS/2% BSA (1:1000, Novus, USA) and incubated with the samples for 1 h at room temperature. Nuclei were stained for 5 min with DAPI (Beyotime, China). Images of axons were obtained using 40 × objectives under a Zeiss LSM 880 confocal microscope (Zeiss, Germany) in a cooler environment. Images were processed using ImageJ (FIJI) software.

### Cell Transfection

Small interfering RNA (siRNA) targeting APJ (siRNA-APJ, sequence: CTGACATGTTACTTCTTCA), siRNA-APJ negative control (NC), miR-124-3p mimic, NC mimic, miR-124-3p inhibitor, and NC inhibitor were synthesized by RiboBio Co. (Guangzhou, China). CTDSP1 overexpression adenovirus and the CTDSP1 overexpression adenovirus negative control (NC) were synthesized by GeneChem. (Shanghai, China). Neurons were transfected with siRNA-APJ, siRNA-APJ NC, miR-124-3p mimic, NC mimic, miR-124-3p inhibitor, NC inhibitor, CTDSP1 overexpression adenovirus, or CTDSP1 overexpression adenovirus NC using transfection reagent according to the manufacturer’s instructions (RiboBio Co, China). During this period, neurons were incubated in culture medium without the penicillin/streptomycin solution.

### Bioinformatics Prediction

The miRNA target gene prediction websites TargetScan (https://www.targetscan.org/vert_80/) was used to predict the target genes of miR-124-3p.

### Dual-Luciferase Reporter Assay

A wild-type (WT) fragment of the 3ʹUTR of CTDSP1 that was predicted to interact with miR-124-3p and a mutant (MUT) fragment of the 3ʹUTR of CTDSP1 were amplified and subcloned into the GV272 vector (GeneChem, Shanghai, China). The constructed luciferase reporter plasmids were named CTDSP1-3′UTR WT and CTDSP1-3′UTR MUT, respectively. Briefly, HEK-293 T cells were seeded in 24-well plates before transfection. When the cells reached 70–80% confluence, cotransfection with either miR-124-3p mimics or miR-NC and either the WT or MUT reporter plasmid was performed using X-tremeGENE HP (ROCHE, Switzerland). After transfection for 48 h at 37 °C, the cells were collected, and luciferase activity was analyzed using a dual-luciferase reporter assay system (Promega Corporation, USA).

### Reverse Transcription-Quantitative Polymerase Chain Reaction (RT‒qPCR)

Total RNA was extracted from cells using TRIzol reagent (Sigma, USA) and quantified using a NanoDrop spectrophotometer (Thermo Fisher Scientific, USA). Next, 1000-ng RNA was reverse transcribed into cDNA using a reverse transcription kit (RR047A, TaKaRa, Japan) according to the manufacturer’s instructions. Then, RT‒qPCR was performed using TB Green Premix Ex TaqTM (RR820A, TaKaRa, Japan) on an Applied Biosystems 7300/7500 Fast real-time PCR system (Roche, CHE). Endogenous expression levels of U6 small nuclear RNA (U6) and GAPDH were used as endogenous controls for normalizing the expression levels of miR-124-3p and its target CTDSP1, respectively. The relative RNA levels were evaluated using the 2^−ΔΔCt^ method.

In the present study, the sequences of the primers that were used for RT‒qPCR were as follows:

miR-124-3p-F: 5ʹ-GCCGAGTAAGGCACGCGGTGAATG-3ʹ;
miR-124-3p-R: 5ʹ-GTCGTATCCAGTGCAGGGTCCG-3ʹ.
U6-F: 5ʹ-CTCGCTTCGGCAGCACA-3ʹ;
U6-R: 5ʹ-AACGCTTCACGAATTTGCGT-3ʹ.
CTDSP1-F: 5ʹ-ATgAgTTCCTACAgCgAATggg-3ʹ;
CTDSP1-R: 5ʹ-AgACACACgACTCTCgAAACAg-3′.
GAPDH-F: 5ʹ-gTggACCTCATggCCTACAT-3ʹ;
GAPDH-R: 5ʹ-TgTgAgggAgATgCTCAgTg-3ʹ.

### Western Blotting

Neurons were washed with PBS and lysed with RIPA lysis buffer (Beyotime, China) containing a protease and phosphatase inhibitor cocktail (CWBIO, China) for 30 min on ice. The samples in each group were mixed well and centrifuged at 12,000×*g* for 20 min and then, the supernatants of each group were collected. The protein concentration was determined by a bicinchoninic acid (BCA) assay kit (CWBIO, China). After being mixed with SDS sample loading buffer, the protein samples were heated at 100 °C for 10 min. Equal amounts of protein from each sample were separated by 12% SDS-PAGE and were then transferred to a 0.2-μm PVDF membrane (Millipore, USA). Subsequently, the membranes were blocked with blocking solution (5% fat-free milk in 1 × TBST) for 1 h and incubated with primary antibodies at 4 °C overnight. The primary antibodies included antibodies against Bcl-2 (1:500; #3498; Cell Signaling Technology, USA), Bax (1:1000; #2772; Cell Signaling Technology, USA), Cleaved Caspase3 (1:1000; #9664; Cell Signaling Technology, USA), AKT (1:1000; #4691; Cell Signaling Technology, USA), phospho-Akt (Ser473) (1:1000; #4060; Cell Signaling Technology, USA), GAP43 (1:1000; #8945; Cell Signaling Technology, USA), CTDSP1 (1:1000; #10952-1-AP; Proteintech, China), and β-actin (1:1000; #3700; Cell Signaling Technology, USA). The next day, 1 × TBST was used to wash the membranes three times for 10 min each. Then, the membranes were incubated with anti-rabbit IgG, HRP-linked antibody (1:3000, Cell Signaling Technology, USA), or anti-mouse IgG, HRP-linked antibody (1:3000, Cell Signaling Technology, USA) at room temperature for 1 h. Then, the membranes were washed three times with 1 × TBST for 10 min each and then, the bands were incubated with chemiluminescence reagents and detected by the ChemiDoc™ Touch Imaging System (Bio-Rad, USA). The gray value was analyzed using NIH ImageJ (FIJI) software.

### Statistical Analysis

All the data were analyzed and graphed with Prism 9.0 (GraphPad Software Inc. La Jolla, CA, USA). Each experiment was repeated at least three times. Normal distribution was assessed for each dataset using the Shapiro–Wilk test. Variance homogeneity was assessed for each dataset using the F test and Brown–Forsythe test or Bartlett’s test (Supplementary Table 1). When normal distribution and homogeneity uniformity assumptions were met, we performed parametric tests, and the data were showed as mean ± standard deviation (mean ± SD). Otherwise, we performed non-parametric tests, and the data were reported as median and interquartile range. Student’s *t* test was used to analyze differences between two groups. One-way ANOVA with Dunnett’s post hoc test was used for multiple group comparisons. *P* value < 0.05 was considered statistically significant.

## Results

### ELA Alleviates OGD-Induced Neuronal Apoptosis and Axonal Damage in an APJ-Dependent Manner

**Fig. 1:**
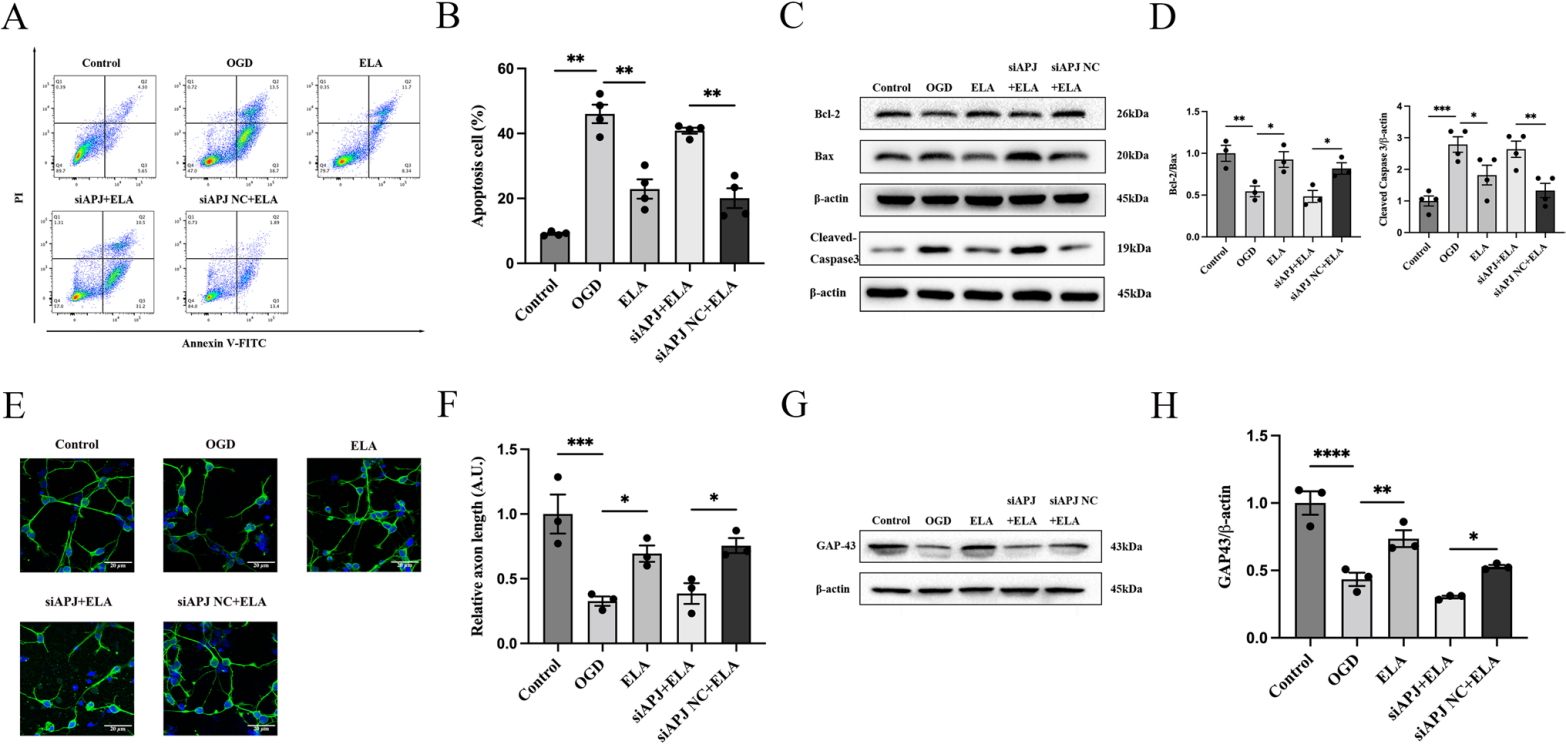
ELA alleviates OGD-induced neuronal apoptosis and axonal damage in an APJ-dependent manner. **A** Neuronal apoptosis results obtained from flow cytometry of various experimental groups. **B** Apoptotic cell rates are statistically presented as median and interquartile range (*n* = 4). **C** Representative blots of Bcl-2, Bax, and  Cleaved Caspase3 protein expression. β-actin was used as a loading control. **D** Quantitative analysis of the Bcl-2/Bax ratio and the Cleaved Caspase3/β-actin ratio. The data are expressed as mean ± SD (Bcl-2/Bax ratio, *n* = 3; Cleaved Caspase3/β-actin, *n* = 4). **E** The length of axons was determined by immunofluorescence staining. β-tubulin III was used to stain axons (green), DAPI was used to stain cell nuclei (blue), and representative confocal images are shown. Scale bars, 20 µm (magnification, 40 ×). **F** Quantification of axon length. The data are presented as mean ± SD. (*n* = 3 independent experiments; at least 30 neurons were analyzed in each experiment per group). **G** Representative blots of GAP43 expression. **H** Quantitative analysis of GAP43 expression levels. The data are presented as mean ± SD (*n* = 3). *P < 0.05, **P < 0.01, ***P < 0.001, and ****P < 0.0001 vs. the respective control. *ELA* Elabela, *OGD* oxygen–glucose deprivation, *siAPJ* small interfering apelin receptor (Colour figure online)

First, we established an in vitro OGD model using cultured primary cortical neurons to simulate IS. Flow cytometry was performed to identify the effect of ELA on neuronal apoptosis. The results showed that the percentage of apoptotic cells among the OGD-treated neurons was markedly higher than that in the control group (*P* < 0.01; Fig. 1A and B). When treated with ELA, the percentage of apoptotic cells significantly decreased compared to that in the OGD group (*P* < 0.01; Fig. 1A and B), indicating that ELA could inhibit apoptosis in neurons. However, the cell apoptosis rate in the siAPJ + ELA group was increased compared to that in the siAPJ NC + ELA group (*P* < 0.01; Fig. 1A and B), and no distinct difference was observed between the OGD group and siAPJ + ELA group (*P* > 0.05; Fig. 1A and B). Moreover, we found that the OGD group had a decrease in the Bcl-2/Bax ratio (*P* < 0.01; Fig. 1C and D) and an increase in the Cleaved Caspase3 expression level (*P* < 0.001; Fig. 1C and D). After ELA treatment, the Bcl-2/Bax ratio was increased, and the expression level of Cleaved Caspase3 was decreased, suggesting that ELA enhanced the expression of antiapoptotic factors and inhibited the expression of proapoptotic factors (*P* < 0.05; Fig. 1C and D). Moreover, when the siAPJ + ELA group was compared with the siAPJ NC + ELA group, the results of the siAPJ + ELA group were consistent with the OGD group (*P* < 0.05; Fig. 1C and D).

Immunofluorescence and western blotting were performed to determine the effect of ELA on the axon growth of neurons. We demonstrated that ELA treatment increased the axon length of neurons (*P* < 0.05; Fig. 1E and F) and upregulated the expression level of GAP43 (*P* < 0.01; Fig. 1G and H). However, when compared to the siAPJ NC + ELA group, the siAPJ + ELA group displayed a decreased axon length and downregulated expression of GAP43 (*P* < 0.05; Fig. 1E–H). No distinct difference was observed between the OGD group and siAPJ + ELA group (*P* > 0.05; Fig. 1E–H).

Taken together, these results showed that ELA may alleviate OGD-induced neuronal injury by inhibiting cell apoptosis and promoting axonal growth and that ELA might not protect neurons with APJ deficiency.

### MiR-124-3p is Expressed at Low Levels in IS, While CTDSP1, Which is a Downstream Target Gene of miR-124-3p, is Highly Expressed in IS

**Fig. 2:**
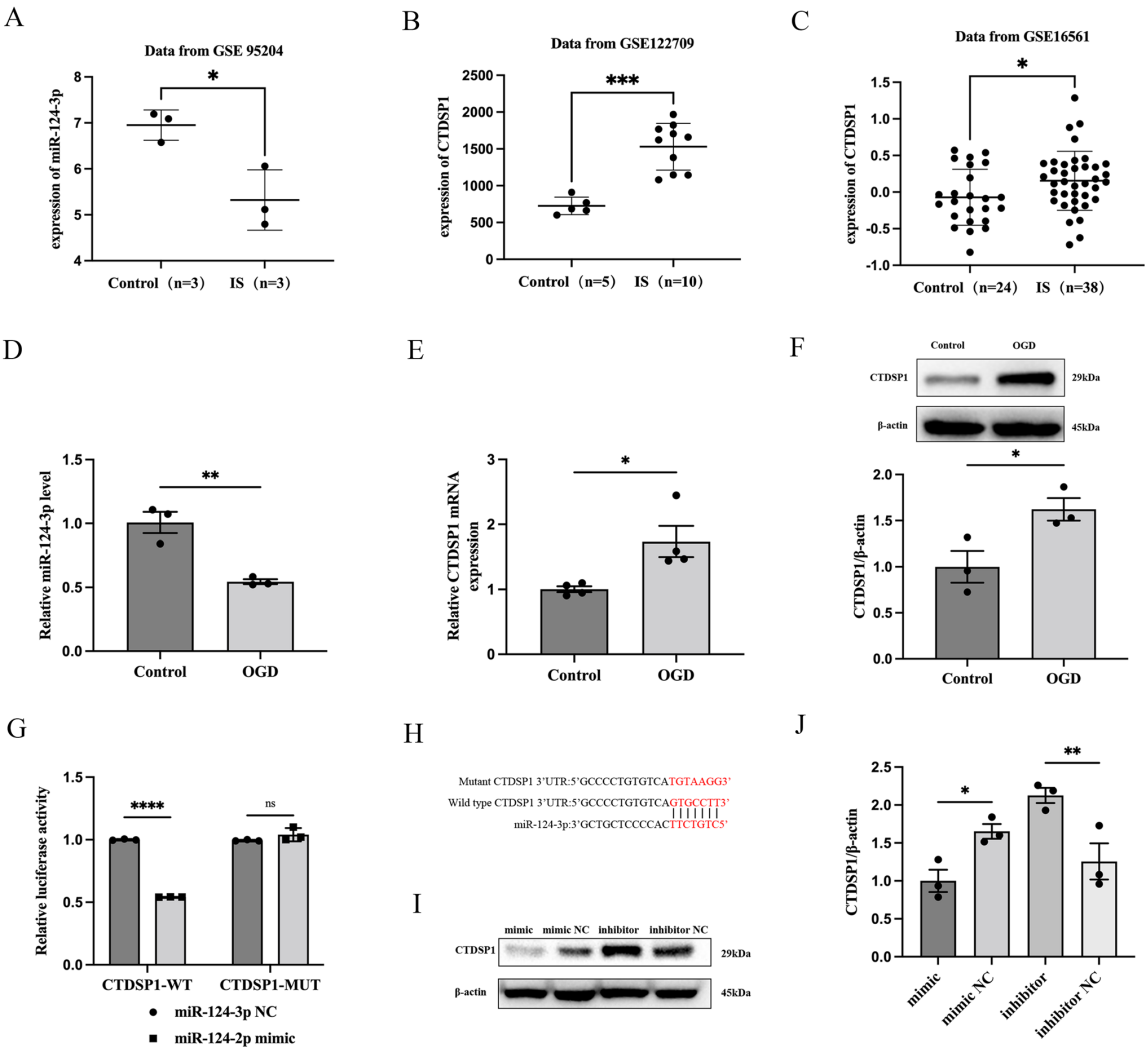
miR-124-3p is expressed at low levels in IS, while CTDSP1, which is a downstream target gene of miR-124-3p, is highly expressed in IS. **A** miR-124-3p expression in patients with IS compared to that in the control group according to the GEO database. Data are shown as mean ± SD (*n* = 3). **B**, **C** The expression of CTDSP1 in patients with IS compared to the control group according to the GEO database. Data are shown as mean ± SD. **D** miR-124-3p expression in OGD-exposed neurons. **E**, **F** The expression level of CTDSP1 under OGD conditions was verified at the protein and mRNA levels. **G** Dual-luciferase reporter assay results. **H** Predicted sites at which miR-124-3p binds to the 3ʹ-UTR of CTDSP1. **I**, **J** Representative blots and western blotting analysis of the CTDSP1 levels in the different groups. β-actin was used as a loading control. **A**–**G** and **J**, mean ± SD (**B** and **C**, *n* > 3; others *n* = 3). *P < 0.05, **P < 0.01, ***P < 0.001, and ****P < 0.0001 vs. the respective control. *RT‒qPCR* reverse transcription-quantitative polymerase chain reaction, *WT* wild-type, *MUT* mutant-type, *UTR* untranslated region, *mimic* miR-124-3p mimic, *mimic NC* miR-124-3p mimic negative control, *inhibitor* miR-124-3p inhibitor, *inhibitor NC* miR-124-3p inhibitor negative control

Next, we wanted to explore the underlying molecular mechanisms by which the ELA/APJ axis exerts its neuroprotective effect. The data from GEO (accession number: GSE95204), collected from samples from IS patients and samples from healthy people, were selected for analysis. As shown in Fig. 2A, we found that miR-124-3p was expressed at lower levels in the IS group than in the control group (*P* < 0.05; Fig. 2A). It is well known that miRNAs usually exert downstream effects through their target genes. We then predicted the target genes of miR-124-3p using TargetScan, and the result strongly suggested that CTDSP1 was the most likely target gene of miR-124-3p. Subsequently, IS data were downloaded from the GEO database (accession numbers: GSE122709 and GSE16561) and used to analyze the differences in CTDSP1 expression. We found that the expression of CTDSP1 was significantly increased in IS patients compared with healthy people and that miR-124-3p followed the opposite trend (*P* < 0.05; Fig. 2B and C). These results suggested that expression of both miR-124-3p and CTDSP1 might be altered during IS, and that miR-124-3p may negatively regulate CTDSP1, which might be the target gene of miR-124-3p. Then, in vitro, we established an OGD model with primary neurons to simulate IS and verified the expression of miR-124-3p and CTDSP1 during OGD. The in vitro results were consistent with those from the database (*P* < 0.05; Fig. 2D–F). Finally, we verified the site at which miR-124-3p directly binds to CTDSP1 by dual-luciferase reporter assay. It was found that miR-124-3p can significantly reduce the fluorescence intensity of the WT group (*P* < 0.0001; Fig. 2G and H) without affecting that of the MUT group (*P* = 0.8471; Fig. 2G and H), which indicated that miR-124-3p directly targets CTDSP1. Additionally, we performed a western blotting assay to further measure the regulatory effect of miR-124-3p on CTDSP1. The results showed that upregulation of miR-124-3p caused a significant decrease in the protein levels of CTDSP1 (*P* < 0.05; Fig. 2I and J); simultaneously, inhibition of miR-124-3p significantly enhanced CTDSP1 expression (*P* < 0.01; Fig. 2I and J). In summary, these results indicated that miR-124-3p and CTDSP1 are abnormally expressed during ischemia, miR-124-3p is an upstream regulator of CTDSP1, and miR-124-3p negatively regulates CTDSP1 expression in OGD-exposed neurons.

### The ELA/APJ Axis Regulates miR-124-3p and Downstream Signaling Pathways

**Fig. 3:**
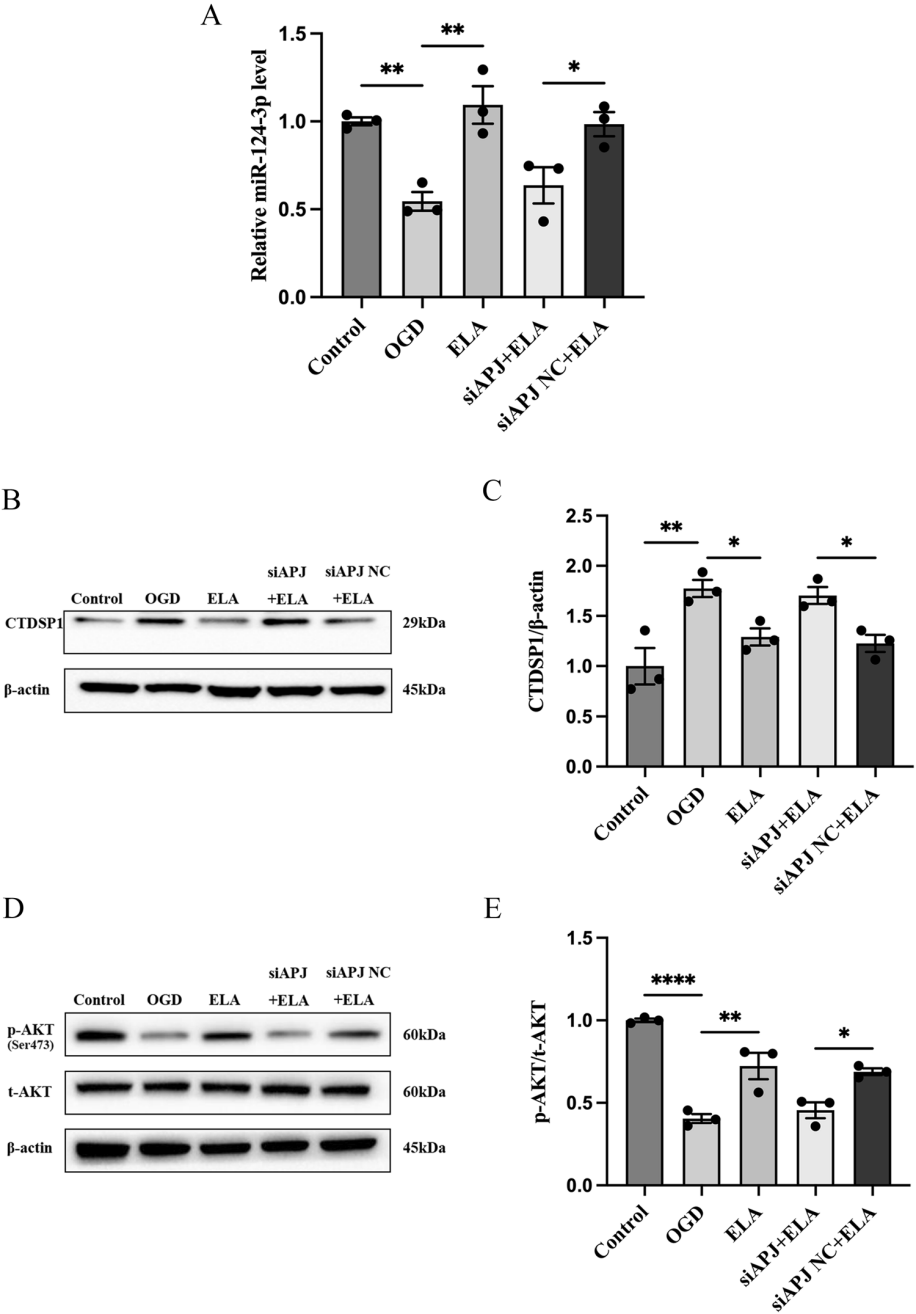
The ELA/APJ axis regulates miR-124-3p and the downstream signaling pathway. **A** Quantification of miR-124-3p expression levels in different experimental groups. The data are shown as mean ± SD (*n* = 3). **B**, **C** Representative blots and quantitative analysis of CTDSP1 expression. β-actin was used as a loading control. The data are presented as mean ± SD (*n* = 3). **D**, **E** Representative blots and quantitative analysis of the p-AKT/t-AKT ratio. The data are expressed as mean ± SD (*n* = 3). *P < 0.05, **P < 0.01, and ****P < 0.0001 vs. the respective control. *ELA* Elabela, *OGD* oxygen–glucose deprivation, *siAPJ* small interfering apelin receptor, *CTDSP*1 C-terminal domain small phosphatase 1, *p-AKT* phosphorylated AKT, *t-AKT* total AKT

After we found that the expression of miR-124-3p and CTDSP1 was altered under ischemia, we wanted to explore whether ELA could regulate miR-124-3p and downstream signaling molecules by activating the APJ receptor. The expression of miR-124-3p was determined by RT‒qPCR, and the expression levels of CTDSP1, p-AKT, and t-AKT were determined by western blotting. The RT‒qPCR results showed that in the OGD group, miR-124-3p was downregulated compared to the control group (*P* < 0.01; Fig. 3A), and miR-124-3p was increased after ELA treatment (*P* < 0.01; Fig. 3A); However, when compared to the siAPJ NC + ELA group, the expression level of miR-124-3p was decreased again in the siAPJ + ELA group (*P* < 0.05; Fig. 3A). Western blotting analysis showed that ELA significantly abrogated the OGD-mediated increase in CTDSP1 expression (*P* < 0.05; Fig. 3B and C) and the decrease in the p-AKT/t-AKT ratio (*P* < 0.01; Fig. 3D and E). However, these results were reversed in the siAPJ + ELA group. These results clearly illustrated that ELA enhances miR-124-3p expression in an APJ-dependent manner and that activation of the ELA/APJ axis might lead to activation of the CTDSP1/AKT signaling pathway.

### MiR-124-3p Mitigates the Neuronal Apoptosis and Axonal Damage Induced by Oxygen–Glucose Deprivation

**Fig. 4:**
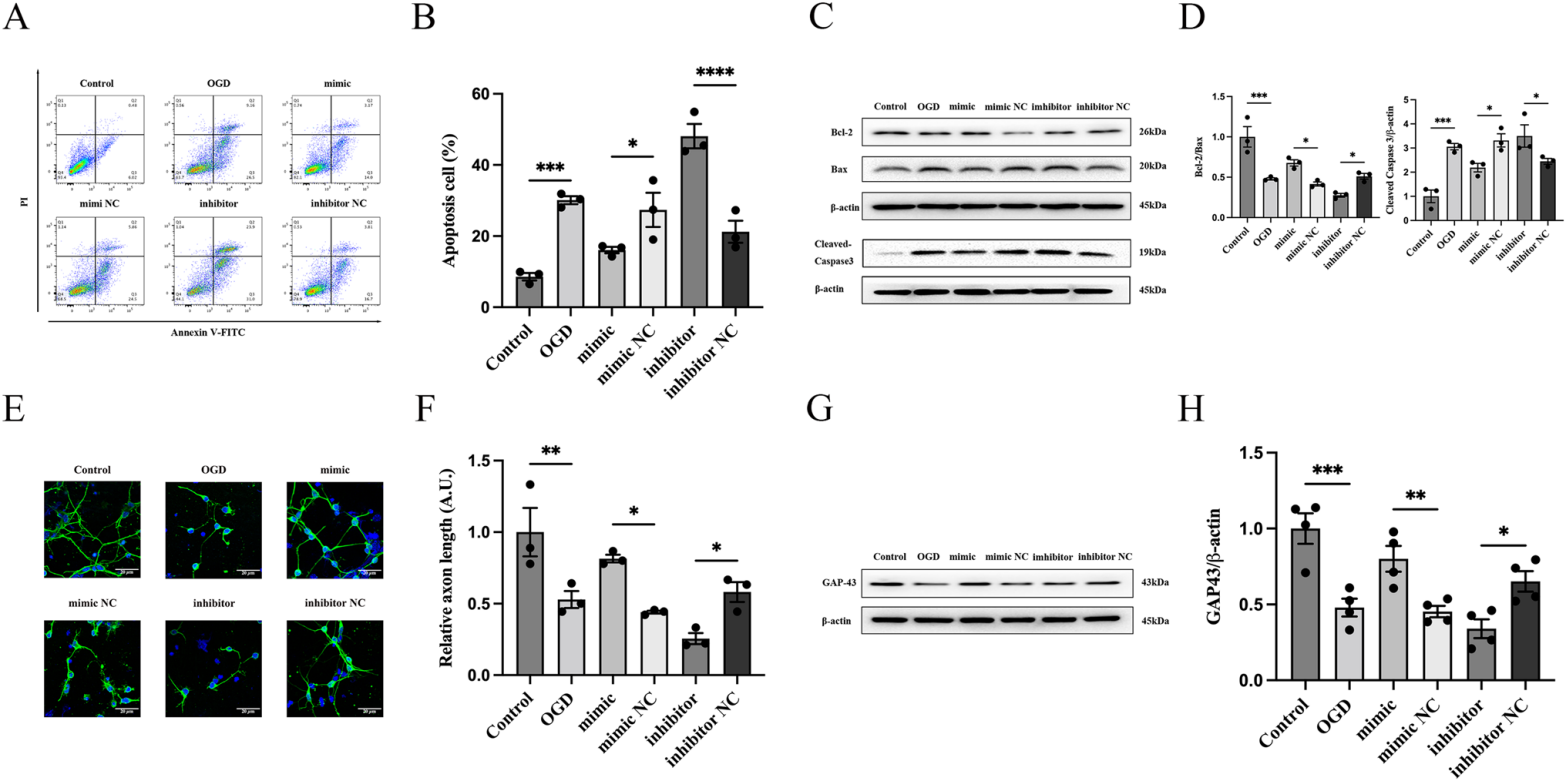
miR-124-3p mitigates the neuronal apoptosis and axonal damage induced by OGD. **A** Neuronal apoptosis results obtained from flow cytometry of various experimental groups. **B** Apoptotic cell rates are statistically presented as mean ± SD (*n* = 3). **C** Representative blots of Bcl-2, Bax, and Cleaved Caspase3 protein expression. β-actin was used as a loading control. **D** Quantitative analysis of the Bcl-2/Bax ratio and Cleaved Caspase3/β-actin ratio. The data are expressed as mean ± SD (*n* = 3). **E** The length of axons was determined by immunofluorescence staining. β-tubulin III was used to stain axons (green), DAPI was used to stain cell nuclei (blue), and representative focal images are shown. Scale bars, 20 µm (magnification, 40 ×). **F** Quantification of axon length. The data are presented as mean ± SD. (*n* = 3 independent experiments; at least 30 neurons were analyzed in each experiment per group). **G** Representative blots of GAP43 expression. **H** Quantitative analysis of GAP43 expression levels. The data are presented as mean ± SD (*n* = 4). *P < 0.05, **P < 0.01, ***P < 0.001, and ****P < 0.0001 vs. the respective control. *ELA* Elabela, *OGD* oxygen–glucose deprivation, *siAPJ* small interfering apelin receptor, *mimic* miR-124-3p mimic, *mimic NC* miR-124-3p mimic negative control, *inhibitor* miR-124-3p inhibitor, *inhibitor NC* miR-124-3p inhibitor negative control (Colour figure online)

Given the findings of the previous experiments, we wanted to further clarify whether miR-124-3p plays a neuroprotective role through inhibiting apoptosis and promoting axon growth in an oxygen–glucose deprivation environment. OGD-exposed neurons were treated with miR-124-3p mimic or miR-124-3p inhibitor. We found that the miR-124-3p mimic dramatically inhibited cell apoptosis (*P* < 0.001; Fig. 4A and B), and the miR-124-3p inhibitor significantly increased cell apoptosis in neurons as shown by a flow cytometry assay (*P* < 0.0001; Fig. 4A and B). To determine whether miR-124-3p affects the axon outgrowth of neurons, an immunofluorescence assay was performed. MiR-124-3p mimic dramatically promoted the axon growth of neurons (*P* < 0.05; Fig. 4E and F), whereas the downregulation of miR-124-3p markedly inhibited axon growth (*P* < 0.05; Fig. 4E and F). Finally, western blotting was used to analyze the expression of pivotal proteins related to apoptosis and axon growth, including Bcl-2, Bax, Cleaved Caspase3, and GAP43. The expression levels of Bcl-2 (an anti-apoptotic protein) and GAP43 (an axon growth-related protein) were markedly increased, whereas those of Bax and Cleaved Caspase3 (pro-apoptotic proteins) were dramatically decreased after transfection with the miR-124-3p mimic (*P* < 0.05; Fig. 4C, D, G, and H). In contrast, changes in the expression levels of Bcl-2, Bax, Cleaved Caspase3, and GAP43 were reversed after transfection with the miR-124-3p inhibitor (*P* < 0.05; Fig. 4C, D, G, and H). In summary, miR-124-3p played a neuron-protective role by suppressing cell apoptosis and enhancing axon growth under OGD conditions.

### ELA Regulates the CTDSP1/AKT Signaling Pathway via miR-124-3p

**Fig. 5:**
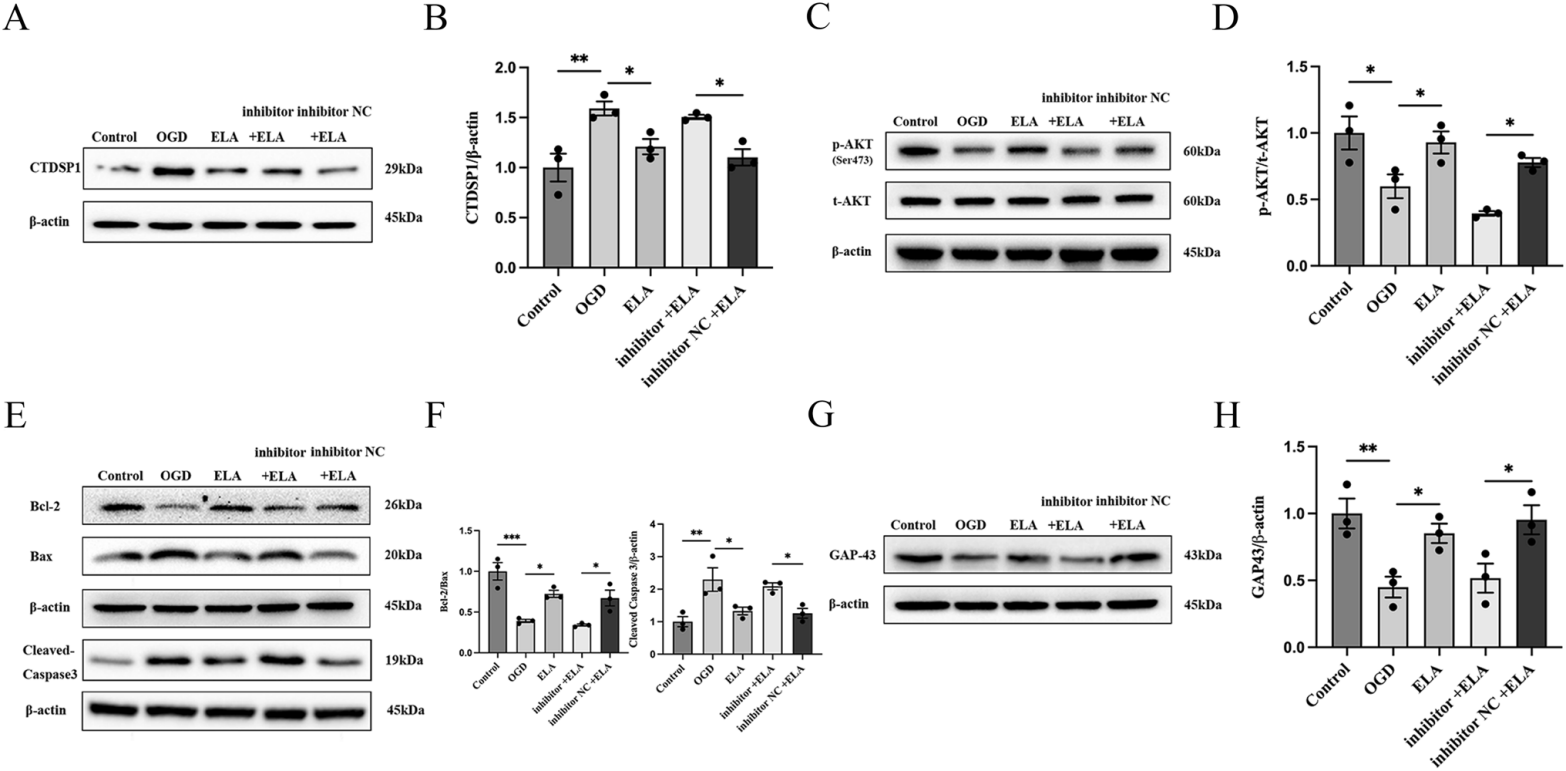
ELA regulates the CTDSP1/AKT pathway via miR-124-3p. **A**, **B** Representative blots and quantitative analysis of CTDSP1 expression. β-actin was used as a loading control. **C**, **D** Representative blots and quantitative analysis of p-AKT and t-AKT expression. β-actin was used as a loading control. **E**, **F** Representative blots and quantitative analysis of Bcl-2, Bax, and Cleaved Caspase3 expression; β-actin was used as a loading control. **G**, **H** Representative blots and quantitative analysis of GAP43 expression. β-actin was used as a loading control. **B**, **D**, **F** and **H**, the data are presented as mean ± SD (*n* = 3). *P < 0.05, **P < 0.01, and ***P < 0.001 vs. the respective control. *ELA* Elabela, *OGD* oxygen–glucose deprivation, *siAPJ* small interfering apelin receptor, *CTDSP1* C-terminal domain small phosphatase 1, *p-AKT* phosphorylated AKT, *t-AKT* total AKT, *inhibitor* miR-124-3p inhibitor, *inhibitor NC* miR-124-3p inhibitor negative control

We verified that ELA and miR-124-3p protect neurons under OGD conditions and that the ELA/APJ axis can activate miR-124-3p and downstream signaling pathways. We wanted to further confirm whether the ELA/APJ axis plays a neuroprotective role by activating the CTDSP1/AKT signaling pathway via miR-124-3p. Western blotting analysis showed that OGD significantly increased the expression level of CTDSP1 (*P* < 0.01; Fig. 5A and B) and decreased the expression level of p-AKT relative to those in the control group (*P* < 0.05; Fig. 5C and D). In the ELA treatment group, the expression level of CTDSP1 was significantly reduced, and the expression level of p-AKT was significantly enhanced compared to those in the OGD group (*P* < 0.05; Fig. 5A–D). Moreover, the inhibitory effects of ELA on CTDSP1 expression and its promoting effect on p-AKT were attenuated by miR-124-3p inhibitor treatment in the inhibitor + ELA groups (*P* < 0.05; Fig. 5A–D). There was no distinct difference observed between the OGD group and inhibitor NC + ELA group (*P* > 0.05; Fig. 5A–D). Moreover, we found that the OGD group showed a decrease in the expression of anti-apoptotic proteins and GAP43 and an increase in the expression level of pro-apoptotic proteins, while ELA treatment reversed these effects (*P* < 0.01; Fig. 5E–H). However, when the miR-124-3p inhibitor + ELA group was compared with the miR-124-3p NC + ELA group, the miR-124-3p inhibitor + ELA group showed consistent results with the OGD group (*P* < 0.05; Fig. 5E–H). Collectively, the results of this study demonstrate that the ELA/APJ axis may play a neuron-protective role by inhibiting apoptosis and axonal damage by activating the CTDSP1/AKT signaling pathway via miR-124-3p.

### MiR-124-3p Alleviates OGD-Induced Apoptosis and Axon Damage in Neurons Through the CTDSP1/AKT Signaling Pathway

**Fig. 6:**
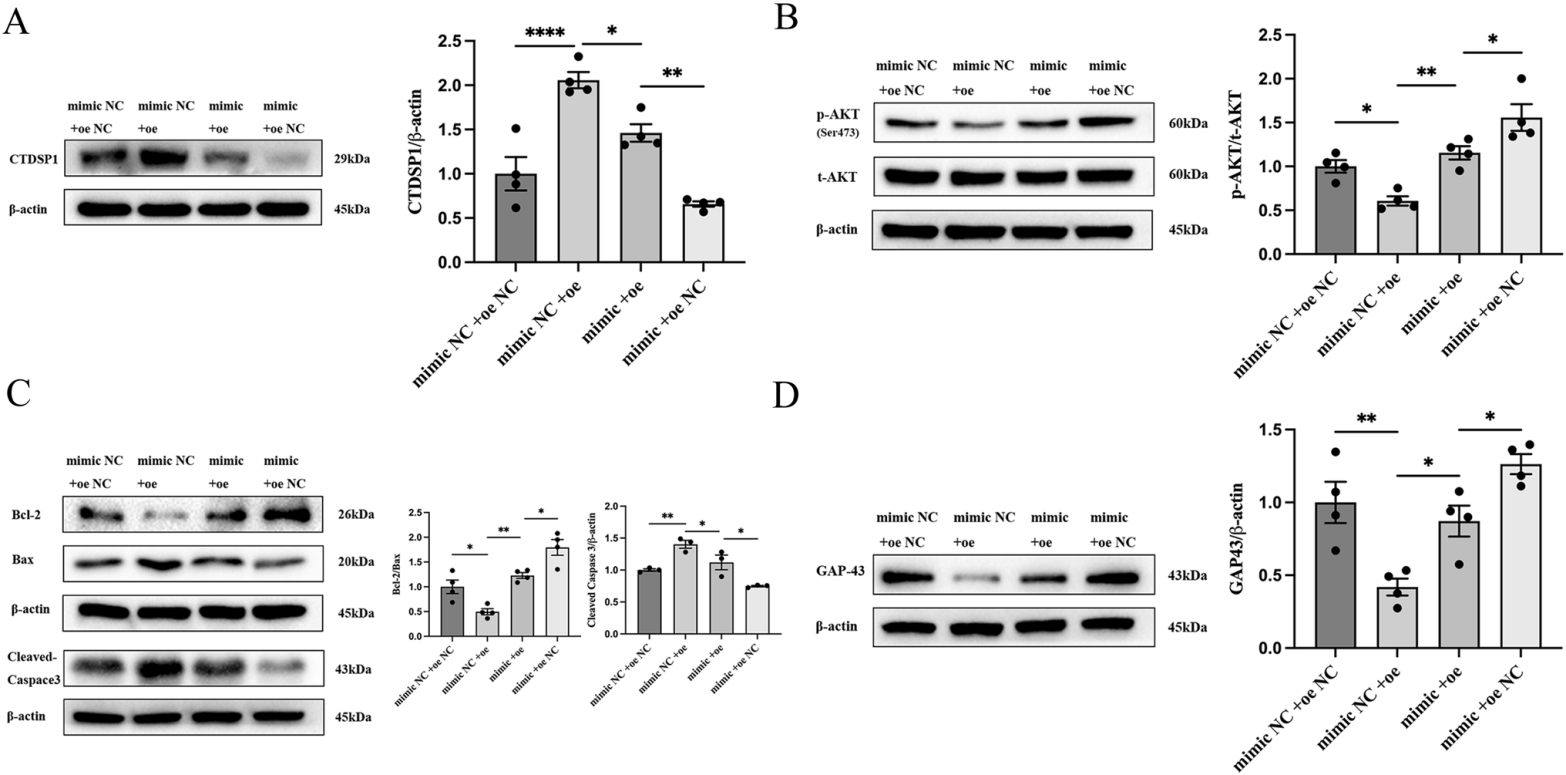
miR-124-3p alleviates OGD-induced apoptosis and axon damage in neurons through the CTDSP1/AKT signaling pathway. Representative immunoblots and quantitative data are provided. **A** CTDSP1 expression in different experimental groups. **B** The p-AKT/t-AKT ratio in different experimental groups. **C** The Bcl-2/Bax ratio and Cleaved Caspase3 expression in different experimental groups **D** GAP43 expression in different experimental groups. **A**–**D** The data are expressed as the mean ± SD (*n* = 4). *P < 0.05, **P < 0.01, and ****P < 0.0001 vs. the respective control. *mimic* miR-124-3p mimic, *mimic NC* miR-124-3p mimic negative control, *oe* overexpression of CTDSP1, *oe NC* overexpression of CTDSP1 negative control, *p-AKT* phosphorylated AKT, *t-AKT* total AKT

We have demonstrated that the ELA/APJ axis regulates the CTDSP1/AKT signaling pathway via miR-124-3p. We wondered whether miR-124-3p promotes the phosphorylation of AKT by regulating CTDSP1. Our Western blotting results demonstrated that CTDSP1 expression was suppressed after miR-124-3p overexpression (miR-124-3p mimic) (*P* < 0.05; Fig. 6A). The total expression level of AKT did not change after alteration of CTDSP1 or miR-124-3p expression; however, the phosphorylation of AKT in the Adv-oeCTDSP1 transfection group was inhibited, and this effect was significantly reversed by miR-124-3p mimic transfection. When Adv-CTDSP1 and miR-124-3p mimic were cotransfected, the level of phosphorylated AKT was higher than that in the Adv-CTDSP1 group but lower than that in the miR-124-3p mimic group (*P* < 0.05; Fig. 6B). Our results demonstrated that miR-124-3p regulates the phosphorylation of AKT by targeting CTDSP1. Furthermore, to clarify whether miR-124-3p alleviates OGD-induced neuronal apoptosis and axon damage through the CTDSP1/AKT signaling pathway, miR-124-3p mimic and CTDSP1 overexpression adenovirus (Adv-CTDSP1) were added. We measured the protein expression levels of Bcl-2, Bax, Cleaved Caspase3, and GAP43 and found them to change in opposite ways in the miR-124-3P mimic and CTDSP1-overexpressing groups. The expression levels of Bcl-2, Bax, Cleaved Caspase3, and GAP43 were similar in the miR-124-3p and CTDSP1 cotransfected group and the miR-124-3p NC and CTDSP1 NC cotransfected group (*P* < 0.05; Fig. 6C and D). These results showed that miR-124-3p inhibits  apoptosis and axonal damage by directly targeting the CTDSP1 3’UTR and downregulating CTDSP expression, thereby activating the phosphorylation of AKT in OGD neurons.

## Discussion

The present study was conducted with the main objective of investigating the beneficial effect of ELA in ischemic stroke in order to develop therapeutic agents. The key and novel findings of the study demonstrated that ELA inhibited neuron apoptosis and alleviated axon damage under OGD conditions. Moreover, the possible mechanism is that ELA plays its role by binding to APJ receptor to upregulate miR-124-3p expression and activate the CTDSP1/AKT signaling pathway.

IS is the leading cause of disability and the second cause of death worldwide (Donkor 2018). At present, major approaches used to treat IS can be divided into two types: recanalization and neuroprotection (Xu et al. 2021). Recanalization is aimed at restoring the blood flow with thrombolytic agents or accessory devices in the acute phase of IS (from minutes to hours) or preventing the reoccurrence of stroke with antiplatelet and anticoagulant agents, while neuroprotection is aimed at protecting neurons from the different pathological factors of IS  (Minnerup et al. 2012). Although recanalization is still the primary treatment for IS, less than 10% of patients benefit clinically due to its narrow therapeutic time window, strict indications, a potential for reperfusion injury, a bleeding risk, and high cost (Mizuma et al. 2018). Hence, there is an urgent need for the continued development of neuroprotective agents. Studies have found that neuroprotective agents alone or in combination with thrombolytic agents can protect brain cells, expand the time window for recanalization treatment, reduce secondary injuries due to reperfusion, and improve neurological function recovery after ischemia (Moretti et al. 2015). In the present study, ELA, as a neuroprotective agent, was demonstrated for the first time to exert neuroprotective effects under oxygen–glucose deprivation condition.

As we all know, a cascade of harmful molecular events is activated in a relatively certain order during IS, which leads to ischemic penumbral neurons undergoing apoptosis and becoming dysfunctional; however, they are salvageable (Xu et al. 2021; Datta et al 2020). In this study, we investigated whether ELA could improve neuronal survival by inhibiting this destructive cascade under hypoxic-ischemic conditions, especially by inhibiting neuronal apoptosis and axonal damage. Our data showed that neurons exhibited a higher apoptosis rate and more severe axon damage in the OGD state. In contrast, ELA-treated neurons presented decreased apoptosis and enhanced axon growth, which indicated that ELA treatment is beneficial for neuron survival during OGD injury. In view of this, we analyzed the possible mechanism of action behind ELA.

It is well known that ELA is a second endogenous peptide ligand of the apelin receptor (APJ). It is similar in structure to apelin but has a higher affinity for the APJ receptor (Zhang et al. 2018). Chng et al. reported that ELA is a hormone essential for heart  development signals via APJ (2013). Our previous study also demonstrated that ELA could inhibit apoptosis in MSCs under hypoxic/ischemic conditions in an APJ-dependent manner (Fu et al. 2020). APJ is widely expressed in the central nervous system, especially in neurons, and studies showed that the apelin/APJ axis exerts neuroprotective effects through diverse pathways during IS, such as anti-oxidative stress, inflammation, and apoptosis (Luo et al. 2020; Tian et al. 2020). In our study, we consistently observed that ELA did not inhibit apoptosis or decrease axonal damage in APJ-silenced neurons, including such aspects as higher apoptosis rate, a decreased Bcl-2/Bax ratio, active state of Cleaved Caspase3, a lower axon growth rate, and a decreased GAP43 expression level. These findings suggested that ELA protects neuron apoptosis and axon damage against OGD injury via the APJ receptor.

Another important and novel finding of this study is that ELA exerts neuroprotective effect is associated with the upregulation of miR-124-3p and activation of the CTDSP1/AKT signaling pathway. MiR-124-3p, which is the most abundant miRNA in brain tissue, has been intensively studied as a diagnostic marker and therapeutic target in IS (Qi et al. 2021; Liu et al. 2015). Clinical studies have shown that the level of miR-124 in the serum of patients with cerebral infarction was significantly reduced within 24 h after ischemia and gradually increased within 48–72 h, and the miR-124 level was also increased on the seventh day after ischemia in patients with IS. However, its level in these patients was still lower than that in healthy people (Weng et al. 2011; Sun et al. 2019). In our study, GEO database analysis showed that the expression of miR-124-3p was decreased in blood samples from patients with acute ischemic stroke at 24 h after onset. And then, our in vitro results showed that miR-124-3p expression decreased in the OGD microenvironment, which is consistent with the results found in the GEO database and previous studies. More importantly, we found that ELA promoted the expression of miR-124-3p under OGD conditions.

In addition, we further explored the protective effect of miR-124-3p against neuronal apoptosis and axonal damage. The data revealed that the overexpression of miR-124-3p decreased OGD-induced apoptosis and axonal damage in neurons, while inhibiting miR-124-3p expression exacerbated neuronal apoptosis and axonal damage. These results are consistent with other studies. For instance, Chen et al. demonstrated that miR-124 extensively participates in the pathophysiology that occurs after IS, functioning as a protector against cerebral ischemia (Chen et al. 2016; Zhang and Meng 2019; Shu and Zhang 2019). Wang et al. observed that miR-124 exerted an antiapoptotic effect in IS by activating the phosphoinositide 3-kinase (PI3K)/protein kinase B (PKB/AKT) signaling pathway, further alleviating cell apoptosis in IS (2017). Furthermore, Sun et al. reported that the upregulation of miR-124 protected neurons against apoptotic cell death in IS by increasing the levels of the anti-apoptotic proteins Bcl-2 and Bcl-xl (2013). Garcia et al. have reported that in SH-SWE neurons, a miR-124 mimic led to neurite outgrowth and mitochondrial activation, whereas a miR-124 inhibitor decreased dendritic spine density (2021). Additionally, Yu et al. found that miR-124 contributes to the control of neurite outgrowth and sustains homeostatic dendritic complexity during neuronal differentiation, neuronal development, and maturation (Yu et al. 2008; Xue et al. 2016).

It is well established that miRNAs regulate target genes by binding to complementary sequences in their 3ʹUTRs, so they function as negative regulators of gene expression (Lu and Rothenberg 2018). Therefore, to explore the underlying mechanisms by which miR-124-3p and its downstream signaling pathway inhibit apoptosis and axonal damage, an exploration of the relationship between miRNAs and their target genes would be helpful. Herein, analysis of data from the GEO database revealed that in the blood samples from patients with acute ischemic stroke, CTDSP1 showed abnormally high expression, in contrast to the low expression of miR-124-3p, suggesting that both miR-124-3p and CTDSP1 may be altered during cerebral ischemia, and that a negative regulatory relationship between them may exist. Subsequently, bioinformatics prediction indicated that CTDSP1 contains  binding sites for miR-124-3p, and we further verified the binding between miR-124-3p and CTDSP1 by dual-luciferase reporter assay. Moreover, western blot analysis showed that transfection of miR-124-3p mimic inhibited CTDSP1 expression, while transfection of miR-124-3p inhibitor increased CTDSP1 expression. Thus, we determined that miR-124-3p is an upstream regulator of CTDSP1 that directly targets CTDSP1 and negatively regulates its expression. Furthermore, we verified that ELA could regulate the expression level of CTDSP1 via APJ and miR-124-3p.

CTDSP1 is an enzyme that removes a phosphate group from phosphorylated proteins (Kohn 2020). Rallabandi et al. reported that CTDSP1 participates in various cellular activities, such as neuronal gene silencing, cell cycle regulation, and some cell signal transduction pathways (Rallabandi et al. 2020). Gervasi et al. found that CTDSP1 knockdown promoted neurotrophic factor expression in both DRG neurons and support cells and promoted DRG neuron axon growth (2021). In the current study, the data showed that treatment with ELA or miR-124-3p mimics enhanced the survival and axon growth of neurons, while overexpression of CTDSP1 partially reversed these effects.

We then focused on the downstream regulation of miR-124-3p and CTDSP1. Studies have described the function of CTDSP1 in dephosphorylation (Burkholder et al. 2018). Here, we reported the effect of miR-124-3p and CTDSP1 on AKT phosphorylation. AKT, which is a serine/threonine kinase, is an intermediate molecule that is involved in many crucial cellular activities, including cell survival, apoptosis, and axon growth. Accumulating data have shown that AKT can be activated through its phosphorylation at Ser473 by a large variety of molecules and relay signals to regulate downstream neuron apoptosis and axon growth (Liu et al. 2022). In our study, CTDSP1 was found to inhibit the phosphorylation of AKT at Ser473, which was consistent with the report that CTDSP1 knockout increased AKT phosphorylation by decreasing the dephosphorylation of AKT at Ser473 (Liao et al. 2017; Zhao et al. 2022). However, this inhibitory effect was reversed by miR-124-3p, which indicated that miR-124-3p significantly increased AKT activation via phosphorylation via CTDSP1 in neurons. On the basis of those findings, our data provided evidence that overexpression of miR-124-3p inhibited apoptosis and decreased axon damage by directly targeting the CTDSP1 3’UTR and downregulating CTDSP1 expression, thereby inducing the phosphorylation of AKT in neurons under OGD. Moreover, we further demonstrated that ELA regulates AKT phosphorylation with the help of APJ and miR-124-3p.

Taken together, the present results are novel and show that ELA administration could reduce neuron apoptosis and axon damage under OGD. This reduction may be attributable to the regulation of the APJ/miR-124-3p/CTDSP1/AKT pathway. However, a limitation of our study is that we did not test whether ELA treatment can improve treatment efficiency in mice with ischemic stroke in vivo, which we may be sought in future.

## Conclusion

To sum up, our research is the first to demonstrate that Elabela protects against apoptosis and axon damage by affecting APJ/miR-124-3p/CTDSP1/AKT pathway-associated proteins, resulting in protective effects on oxygen–glucose deprivation-exposed neurons in vitro. Elabela, which is a novel peptide, provides the basis for exploring drugs for the treatment of ischemic stroke.

## Supplementary Information

Below is the link to the electronic supplementary material.

Supplementary file1 (PDF 532 KB)

Supplementary file2 (PDF 135 KB)

Supplementary file3 (PDF 333 KB)

## Data Availability

All data generated or analyzed during this study are included in this published article.
