# Supplementary material for: Elabela, a Novel Peptide, Exerts Neuroprotective Effects Against Ischemic Stroke Through the APJ/miR-124-3p/CTDSP1/AKT Pathway
Source: Cell Mol Neurobiol. 2023 Apr 27;43(6):2989–3003. doi: 10.1007/s10571-023-01352-6 (PMC10333378; doi:10.1007/s10571-023-01352-6)

Elabela, a novel peptide, exerts neuroprotective effects against ischemic stroke through the APJ/miR-124-3p/CTDSP1/AKT pathway

Kang-long Zhang[1,+], Shuang-mei Li[1, +], Jing-yu Hou[1, +], Ying-hui Hong[1], Xu-xiang Chen[1], Chang-qing Zhou[1], Hao Wu[2], Guang-hui Zheng[2], Chao-tao Zeng[2], Hai-dong Wu[1], Jia-ying Fu[1], Tong Wang[1]*

[1]Department of Emergency, the Eighth Affiliated Hospital of Sun Yat-sen University, Shenzhen, Guangdong, 518003, P. R. China

[2]Department of Emergency, Sun Yat-sen Memorial Hospital of Sun Yat-sen University, Guangzhou, Guangdong, 510120, P. R. China

* **Correspondence**: Prof.Tong Wang，wangtong@mail.sysu.edu.cn

+ Equal contributors

Supplementary S1： Figure S1.Full uncropped blots for Figure1

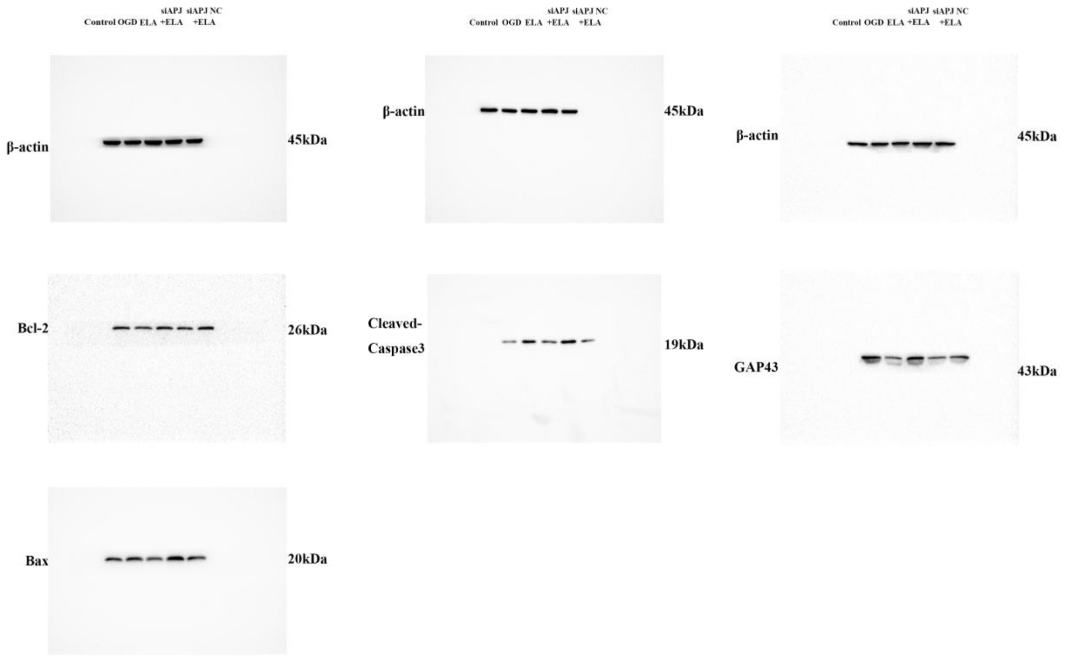

Supplementary S1： Figure S2.Full uncropped blots for Figure2

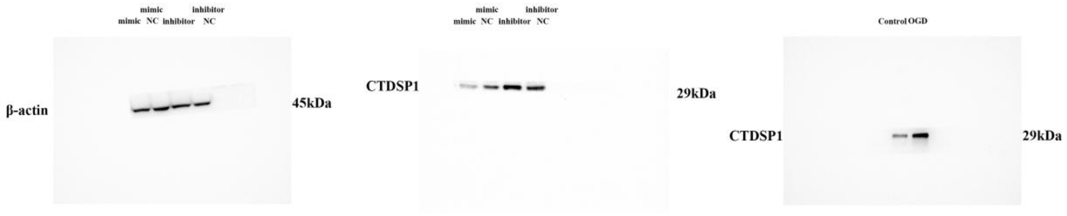

Supplementary S1： Figure S3. Full uncropped blots for Figure3

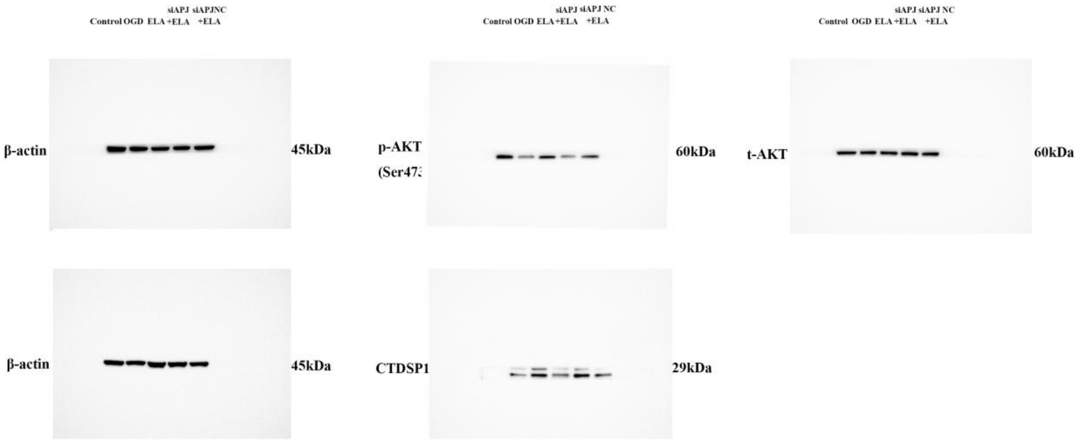

Supplementary S1：Figure S4.Full uncropped blots for Figure4

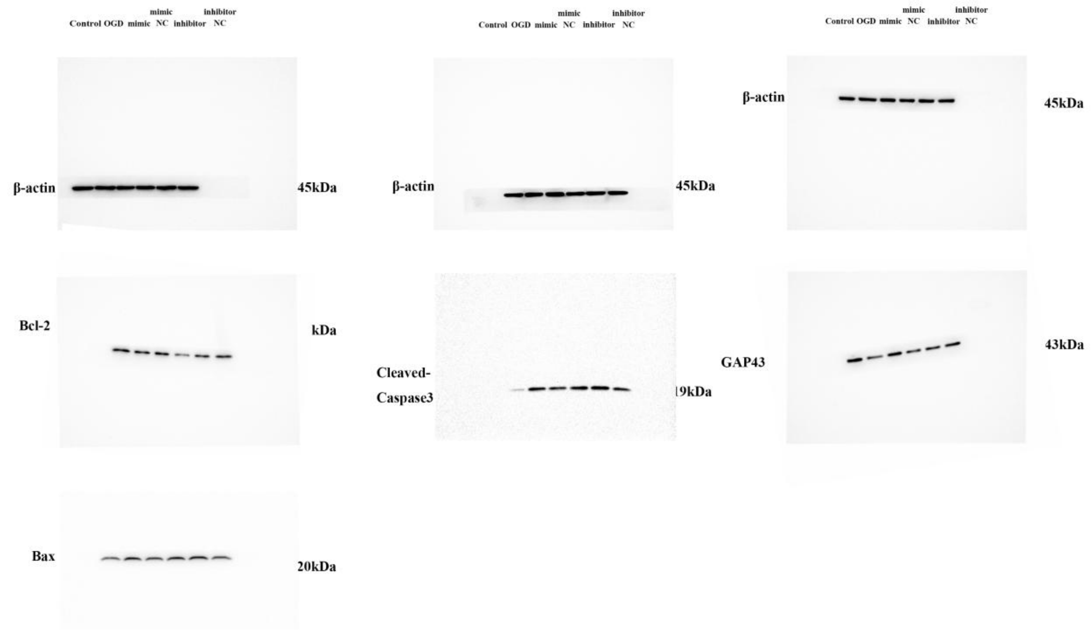

Supplementary S1：Figure S5.Full uncropped blots for Figure5

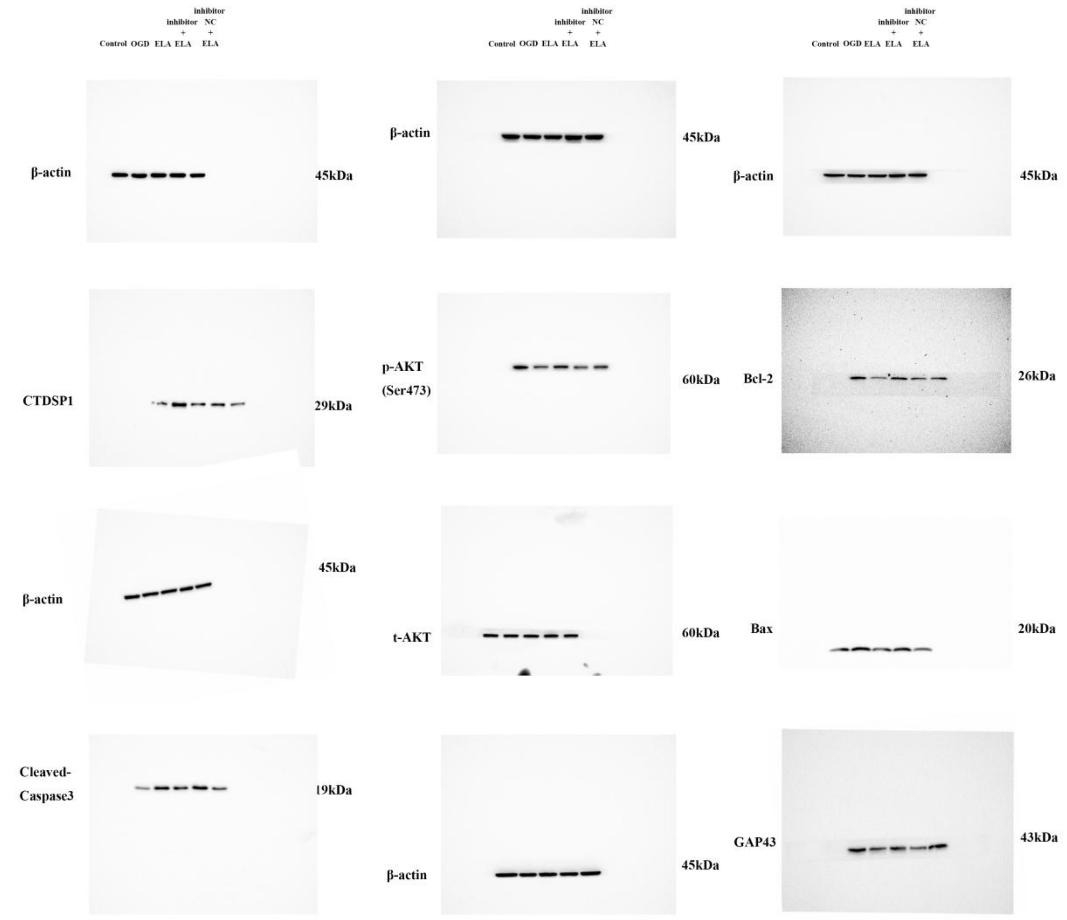

Supplementary S1：Figure S6.Full uncropped blots for Figure6

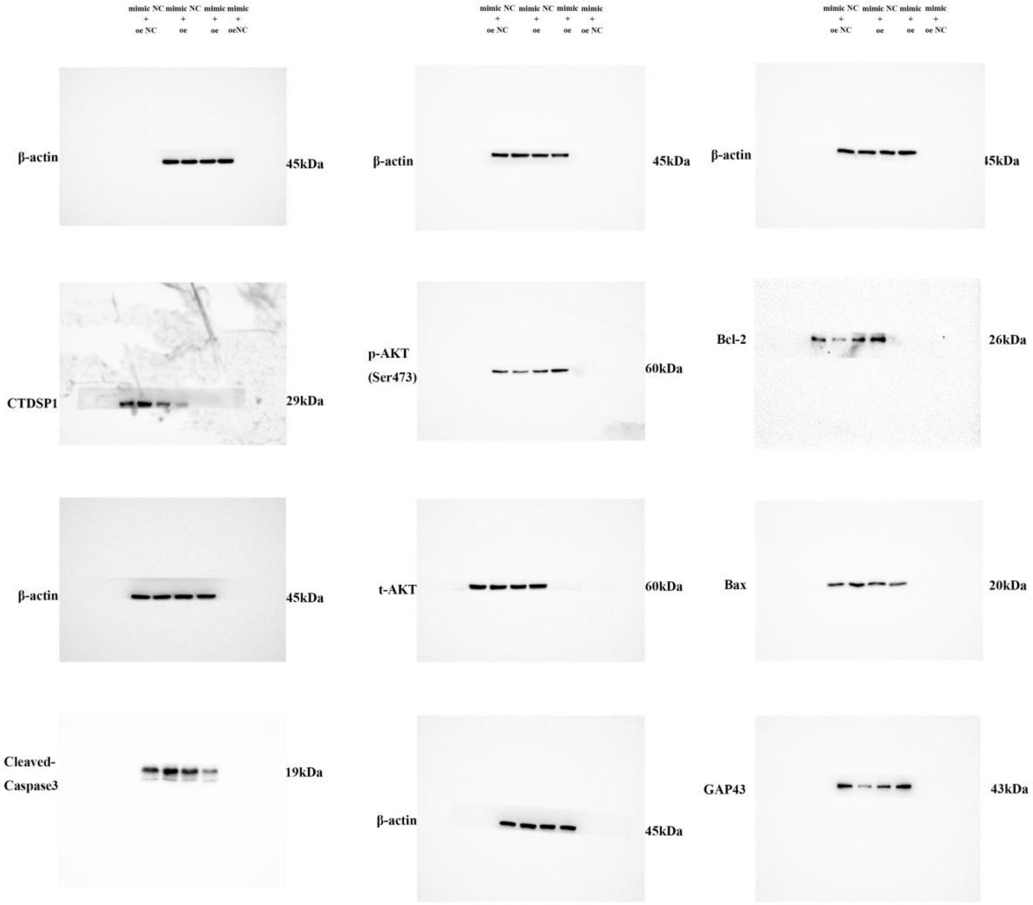

Supplement: Supplementary file 1 — Supplementary file1 (PDF 532 KB) [file 10571_2023_1352_MOESM1_ESM.pdf]
