# Supplementary material for: Elabela, a Novel Peptide, Exerts Neuroprotective Effects Against Ischemic Stroke Through the APJ/miR-124-3p/CTDSP1/AKT Pathway
Source: Cell Mol Neurobiol. 2023 Apr 27;43(6):2989–3003. doi: 10.1007/s10571-023-01352-6 (PMC10333378; doi:10.1007/s10571-023-01352-6)
Supplement: Supplementary file 2 — Supplementary file2 (PDF 135 KB) [file 10571_2023_1352_MOESM2_ESM.pdf]

Elabela, a novel peptide, exerts neuroprotective effects against ischemic stroke through the APJ/miR-124-3p/CTDSP1/AKT pathway

Kang-long Zhang[1,+], Shuang-mei Li[1, +], Jing-yu Hou[1, +], Ying-hui Hong[1], Xu-xiang Chen[1], Chang-qing Zhou[1], Hao Wu[2], Guang-hui Zheng[2], Chao-tao Zeng[2], Hai-dong Wu[1], Jia-ying Fu[1], Tong Wang[1]*

[1]Department of Emergency, the Eighth Affiliated Hospital of Sun Yat-sen University, Shenzhen, Guangdong, 518003, P. R. China

[2]Department of Emergency, Sun Yat-sen Memorial Hospital of Sun Yat-sen University, Guangzhou, Guangdong, 510120, P. R. China

* **Correspondence**: Prof.Tong Wang, wangtong@mail.sysu.edu.cn

+ Equal contributors

Supplementary S2: Primary antibody specific materials

Bcl-2 (D17C4) Rabbit mAb,CST,cat. #3498.the antibody was validated by the CST company using Bcl-2 transfect cell lines,please refer to the manufacture's description: www.cellsignal.cn/products/primary-antibodies/bcl-2-d17c4-rabbit-mab/3498?_=1678081407311&Ntt=3498s&tahead=true

Bax antibody,CST,cat. #2772.the antibody was validated by the company using Bax knock out cell lines,please refer to the manufacture's description: www.cellsignal.cn/products/primary-antibodies/bax-antibody/2772?_=1678080322060&Ntt=2772s&tahead=true

Cleaved Caspase-3 (Asp175) (5A1E) Rabbit mAb, CST,cat.#9664.the antibody was validated by the company using staurosporine #9953 (1uM, 3 hours) or etoposide #2200 (25uM, 5 h) treated C6 (rat), NIH/3T3 (mouse) and Jurkat (human),please refer to the manufacture's description:www.cellsignal.cn/products/primary-antibodies/cleaved-caspase-3-asp175-5a1e-rabbit-mab/9664?site-search-type=Products&N=4294956287&Ntt=9664&fromPage=plp&_requestid=663330

Phospho-Akt (Ser473) (D9E) XP® Rabbit mAb,CST,cat.#4060.the antibody wa-s validated by the company using LY294002/ Wortmannin-treated PC-3 cells a-nd NIH/3T3 cells treated with serum starvation or PDGF,please refer to the m-anufacture'sdescription:www.cellsignal.cn/products/primary-antibodies/phospho-akt-ser473-d9e-xp-rabbit-mab/4060?site-search-type=Products&N=4294956287&Ntt=4060&fromPage=plp&_requestid=752789

GAP43 (D9C8) Rabbit mAb, CST,CAT.#8945.the antibody was validated by the article using GAP-43 knock out cerebellar granule cells,please refer to the manufacture's description: DOI: 10.1007/s12311-008-0049-5

CTDSP1 Polyclonal antibody,proteintech,cat.10952-1-AP,the antibody was validated by the article using CTDSP1 knock out cell lines,please refer to the manufacture's description: DOI: 10.1074/jbc.RA118.004722.
