# Supplementary material for: Elabela, a Novel Peptide, Exerts Neuroprotective Effects Against Ischemic Stroke Through the APJ/miR-124-3p/CTDSP1/AKT Pathway
Source: Cell Mol Neurobiol. 2023 Apr 27;43(6):2989–3003. doi: 10.1007/s10571-023-01352-6 (PMC10333378; doi:10.1007/s10571-023-01352-6)
Supplement: Supplementary file 3 — Supplementary file3 (PDF 333 KB) [file 10571_2023_1352_MOESM3_ESM.pdf]

Elabela, a novel peptide, exerts neuroprotective effects against ischemic stroke through the APJ/miR-124-3p/CTDSP1/AKT pathway

Kang-long Zhang[1,+], Shuang-mei Li[1, +], Jing-yu Hou[1, +], Ying-hui Hong[1], Xu-xiang Chen[1], Chang-qing Zhou[1], Hao Wu[2], Guang-hui Zheng[2], Chao-tao Zeng[2], Hai-dong Wu[1], Jia-ying Fu[1], Tong Wang[1]*

[1]Department of Emergency, the Eighth Affiliated Hospital of Sun Yat-sen University, Shenzhen, Guangdong, 518003, P. R. China

[2]Department of Emergency, Sun Yat-sen Memorial Hospital of Sun Yat-sen University, Guangzhou, Guangdong, 510120, P. R. China

* **Correspondence**: Prof.Tong Wang，wangtong@mail.sysu.edu.cn

+ Equal contributors

Supplementary Table1:for Normality and variance heterogeneity and the statistical analysis performed for each experiment

Fig.number	Groups	Normality	Variance homogeneity	Type of statistical analysis
Figure1B	Control	W = 0.9437; p = 0.6770	Brown-Forythe test	Non-Parametric
	OGD	W = 0.9862; p = 0.9372	F(4,9.631)=42.91,P < 0.0001	
	ELA	W = 0.9820; p = 0.9136		
	siAPJ+ELA	W= 0.8235; p = 0.1514		
	siAPJ NC+ELA	W= 0.8691; p = 0.2941		
Figure1D	Control	W = 0.9671; p = 0.6515	Brown-Forythe test	Parametric
Bcl-2/Bax	OGD	W = 0.9900; p = 0.8088	F(4,10)=0.1053,P=0.9780	
	ELA	W = 0.9742; p = 0.6917		
	siAPJ+ELA	W= 0.9081; p = 0.4116		
	siAPJ NC+ELA	W= 0.8782; p = 0.3193		
Cleaved-Caspase3	Control	W = 0.9000; p = 0.4309	Bartlett's test	Parametric
/β-actin	OGD	W = 0.8301; p = 0.1680	P=0.8806	
	ELA	W = 0.8629; p = 0.2706		
	siAPJ+ELA	W= 0.8633; p = 0.2723		
	siAPJ NC+ELA	W= 0.8304; p = 0.1689		
Figure1F	Control	W = 0.9643; p = 0.6369	Brown-Forythe test	Parametric
	OGD	W = 0.9505; p = 0.5714	F(4,10)=0.7037,P=0.6072	
	ELA	W = 0.9586; p = 0.6086		
	siAPJ+ELA	W= 0.9276; p = 0.4797		
	siAPJ NC+ELA	W= 0.8516; p = 0.2447		
Figure1H	Control	W = 0.8035; p = 0.1228	Brown-Forythe test	Parametric
	OGD	W = 0.9641; p = 0.6361	F(4,10)=0.5367,P=0.7124	
	ELA	W = 0.8027; p = 0.1209		
	siAPJ+ELA	W= 0.7791; p = 0.0655		
	siAPJ NC+ELA	W= 0.9381; p = 0.5200		
Figure2A	Control	W = 0.8702; p = 0.2961	F test	Parametric
（GSE95204）	IS	W = 0.9245; p = 0.4685	F(2,2)=3.963,P=0.4030	
Figure2B	Control	W = 0.9389; p = 0.6581	F test	Parametric

（GSE122709)	IS	W = 0.9036; p = 0.2399	F(9,4)=7.115,P=0.0744	
Figure2C	Control	W = 0.9488; p = 0.2558	F test	Parametric
GSE16561	IS	W = 0.9708; p = 0.4135	F(37,23)=1.109,P=0.8090	
Figure2D	Control	W = 0.8444; p = 0.2255	F test	Parametric
	OGD	W = 0.8372; p = 0.2067	F(2,2)=18.30,P=0.1036	
Figure2E	Control	W = 0.9163; p = 0.5164		Non-Parametric
	OGD	W = 0.7373; p = 0.0292		
Figure2F	Control	W = 0.9834; p = 0.7529	F test	Parametric
	OGD	W = 0.8544; p = 0.2524	F(2,2)=1.983,P=0.6705	
Figure2G	miR-124-3p NC	W = 0.9010; p = 0.3886	F test	Parametric
CTDSP1-WT	miR-124-3p	W = 1.0000; p = 0.9927	F(2,2)=15.80,P=0.1190	
CTDSP1-MUT	miR-124-3p NC	W = 0.8169; p = 0.1555	F test	Parametric
	miR-124-3p	W = 0.8929; p = 0.3631	F(2,2)=1.349,P=0.8513	
Figure2J	mimic	W = 0.9495; p = 0.5671	Brown-Forythe test	Parametric
	mimic Nc	W= 0.9301; p = 0.4890	F(3,8)=0.3225,P=0.8092	
	inhibitor	W= 0.9010; p = 0.3886		
	inhibitor NC	W= 0.8665; p = 0.2857		
Figure3A	Control	W = 0.9892; p = 0.8009	Brown-Forythe test	Parametric
	OGD	W = 0.7814; p = 0.0709	F(4,10)=0.3780,P=0.8194	
	ELA	W= 0.9669; p = 0.6507		
	siAPJ+ELA	W= 0.7854; p = 0.0800		
	siAPJ NC+ELA	W= 0.9483; p = 0.5618		
Figure3C	Control	W = 0.8710; p = 0.2983	Brown-Forythe test	Parametric
	OGD	W = 0.9643; p = 0.6369	F(4,10)=0.2699,P=0.8908	
	ELA	W= 0.9440; p = 0.6012		
	siAPJ+ELA	W= 0.7718; p = 0.3188		
	siAPJ NC+ELA	W= 0.9332; p = 0.5663		
Figure3E	Control	W = 0.9995; p = 0.9586	Brown-Forythe test	Parametric
	OGD	W = 0.9883; p = 0.7935	F(4,10)=0.4362,P=0.7798	
	ELA	W= 0.7808; p = 0.0694		
	siAPJ+ELA	W= 0.7803; p = 0.0682		

	siAPJ NC+ELA	W= 0.9998; p = 0.9755		
Figure4B	Control	W = 0.8765; p = 0.3142	Brown-Forythe test	Parametric
	OGD	W = 0.9868; p = 0.7804	F(5,12)=0.8845,P=0.5206	
	mimic	W = 0.8515; p = 0.2446		
	mimic Nc	W= 1.0000; p = 0.9887		
	inhibitor	W= 0.8558; p = 0.2562		
	inhibitor NC	W= 0.9250; p = 0.4701		
Figure4D	Control	W = 0.9566; p = 0.5990	Brown-Forythe test	Parametric
Bcl-2/Bax	OGD	W = 0.8507; p = 0.2424	F(5,12)=1.450,P=0.2763	
	mimic	W = 0.9916; p = 0.8246		
	mimic Nc	W= 0.9420; p = 0.5353		
	inhibitor	W= 0.9443; p = 0.5451		
	inhibitor NC	W= 0.8680; p = 0.2897		
Cleaved-Caspase3	Control	W = 0.8057; p = 0.1281	Brown-Forythe test	Parametric
/β-actin	OGD	W = 0.9877; p = 0.7874	F(5,12)=0.2945,P=0.9069	
	mimic	W = 0.8498; p = 0.2399		
	mimic Nc	W= 0.9601; p = 0.6158		
	inhibitor	W= 0.7812; p = 0.0704		
	inhibitor NC	W= 0.9819; p = 0.7425		
Figure4F	Control	W = 0.8929; p = 0.3631	Brown-Forythe test	Parametric
	OGD	W = 0.9397; p = 0.5261	P=0.4912	
	mimic	W = 0.9231; p = 0.4633		
	mimic Nc	W= 1.9643; p = 0.6369		
	inhibitor	W= 0.8176; p = 0.1572		
	inhibitor NC	W= 0.8596; p = 0.2665		
Figure4H	Control	W = 0.8081; p = 0.1176	Brown-Forythe test	Parametric
	OGD	W = 0.9921; p = 0.9680	F(5,18)=0.6144,P=0.6903	
	mimic	W = 0.9434; p = 0.6749		
	mimic Nc	W= 0.8700; p = 0.2977		
	inhibitor	W= 0.8911; p = 0.3880		
	inhibitor NC	W= 0.8697; p = 0.2964		

Figure5B	Control	W = 0.8929; p = 0.3631	Brown-Forythe test	Parametric
	OGD	W = 0.8791; p = 0.2612	F(4,10)=0.4668,P=0.7591	
	ELA	W= 0.9689; p = 0.6614		
	inhibitor+ELA	W= 0.9796; p = 0.7262		
	inhibitor NC+ELA	W= 0.8748; p = 0.3093		
Figure5D	Control	W = 0.9956; p = 0.8734	Brown-Forythe test	Parametric
	OGD	W = 0.9082; p = 0.4122	F(4,10)=0.9186,P=0.4902	
	ELA	W= 0.9740; p = 0.6908		
	inhibitor+ELA	W= 0.9998; p = 0.9750		
	inhibitor NC+ELA	W= 0.9815; p = 0.7393		
Figure5F	Control	W = 0.9316; p = 0.4948	Brown-Forythe test	Parametric
Bcl-2/Bax	OGD	W = 0.9972; p = 0.8988	F(4,10)=1.122,P=0.3991	
	ELA	W= 0.7786; p = 0.0643		
	inhibitor+ELA	W= 0.9962; p = 0.8822		
	inhibitor NC+ELA	W= 0.9978; p = 0.9113		
Cleaved-Caspase3	Control	W = 0.9614; p = 0.6221	Brown-Forythe test	Parametric
/β-actin	OGD	W = 0.8375; p = 0.2076	F(4,10)=0.4298,P=0.7842	
	ELA	W= 0.8564; p = 0.2578		
	inhibitor+ELA	W= 0.8048; p = 0.1261		
	inhibitor NC+ELA	W= 0.9967; p = 0.8907		
Figure5H	Control	W = 0.9249; p = 0.4699	Brown-Forythe test	Parametric
Bcl-2/Bax	OGD	W = 0.9525; p = 0.5805	F(4,10)=0.1116,P=0.9756	
	ELA	W= 0.9999; p = 0.9847		
	inhibitor+ELA	W= 0.9976; p = 0.9073		
	inhibitor NC+ELA	W= 0.9648; p = 0.6369		
Figure6A	mimic NC+oe NC	W = 0.9482; p = 0.7074	Bartlett's test	Parametric
	mimicNC +oe	W = 0.8302; p = 0.1682	P=0.0808	
	mimic +oe	W = 0.7909; p = 0.0869		
	mimic+oe NC	W= 0.8615; p = 0.2658		
Figure6B	mimic NC+oe NC	W = 0.9685; p = 0.8320	Bartlett's test	Parametric
	mimicNC +oe	W = 0.8959; p = 0.4109	P=0.3274	

	mimic +oe	W = 0.9664; p = 0.8189		
	mimic+oe NC	W= 0.8087; p = 0.1189		
Figure6C	mimic NC+oe NC	W = 0.9960; p = 0.9857	Bartlett's test	Parametric
Bcl-2/Bax	mimicNC +oe	W = 0.9351; p = 0.6248	P=0.2718	
	mimic +oe	W = 0.9234; p = 0.5560		
	mimic+oe NC	W= 0.9120; p = 0.4928		
Cleaved-Caspase3	mimic NC+oe NC	W = 0.9998; p = 0.9733	Brown-Forythe test	Parametric
/β-actin	mimicNC +oe	W = 0.9153; p = 0.4359	P=0.3710	
	mimic +oe	W = 0.9299; p = 0.4882		
	mimic+oe NC	W= 0.8815; p = 0.3288		
Figure6D	mimic NC+oe NC	W = 0.9988; p = 0.9965	Bartlett's test	Parametric
	mimicNC +oe	W = 0.9452; p = 0.6861	P=0.4706	
	mimic +oe	W = 0.9108; p = 0.4866		
	mimic+oe NC	W= 0.8958; p = 0.4105		
